# Concerning New York Milk

**Published:** 1854-12

**Authors:** 


### CONCERNING NEW YORK MILK.

The New York Mirror says that, early in the morning from thirty to forty milkmen, with "Orange county milk," and other titles "tasting of Flora and the country green," blazoned on their wagons, may be seen filling their cans with water from a pump in Sixteenth-street, near tenth avenue. After the water is put into the cans, he says a yellowish powder is also put in, and the whole well shaken, in order to give the milk a rich creamy look; and those early boys don't seem to make any secret of the thing; it is all in the way of trade.

A most rigid surveillance is kept up in Paris, and in all parts of the country from whence the capital is supplied, over the milk which is forwarded for the consumption of its inhabitants. Thirteen farmers have just been condemned to fines of one hundred francs and under, and one to eight days' imprisonment, for sending milk mixed with water.

While at our house on the Hudson last summer, we used a medical guage in testing the purity of unsophisticated, unadulterated milk just from an excellent cow, and allowed to cool; it gave a density of *forty;* pure well water at our door gave *five* only, showing that there was seven times more substance in pure milk than in pure water. On coming to town we thought it of high importance to get as good a quality of milk as possible, and for that purpose obtained milk from different wagons; some had so much water and so little cream, that it wouldn't boil, consequently the mercury ballasted glass tube ranged at all distances between five and forty; one man's dairy gave a measure of *forty-five*, thus containing one-eighth more substance than milk known to be fresh and pure; as it kept well in midsummer all day, gave no sediment, boiled easily, and chemical tests did not indicate any foreign admixture, we concluded it was purer milk than we had in the country, in consequence of the evaporation of the more watery particles in warm weather, from the time of milking to the time of measurement some six or eight hours, as it came to the city by the H. R. Railroad. So, without his knowledge or consent, and without hope of reward, we give our milkman's name—P. J. Gurnie, 287 West 24th-street, New York. We trust, however, he will feel in gratitude bound, should he ever see this, as unfortunately for himself he is not a subscriber to the "Journal," to not keep our little Bob until eight o'clock every morning waiting for his breakfast, his former supply having been lately monopolized by a stranger.

